# Vegetative Insecticidal Protein Vip3Aa Is Transported via Membrane Vesicles in *Bacillus thuringiensis* BMB171

**DOI:** 10.3390/toxins14070480

**Published:** 2022-07-13

**Authors:** Yizhuo Zhang, Xuelian Li, Hongwei Tian, Baoju An, Bing Yan, Jun Cai

**Affiliations:** 1 Department of Microbiology, College of Life Sciences, Nankai University, Tianjin 300071, China; 2120201040@mail.nankai.edu.cn (Y.Z.); 2120201043@mail.nankai.edu.cn (X.L.); 2120211166@mail.nankai.edu.cn (H.T.); 1120190491@mail.nankai.edu.cn (B.A.); iceyan@nankai.edu.cn (B.Y.); 2 Key Laboratory of Molecular Microbiology and Technology, Ministry of Education, Tianjin 300071, China; 3 Tianjin Key Laboratory of Microbial Functional Genomics, Tianjin 300071, China

**Keywords:** *Bacillus thuringiensis*, Vip3Aa, membrane vesicles, protein secretion

## Abstract

Vegetative insecticidal protein Vip3Aa, secreted by many *Bacillus thuringiensis* (Bt) strains during the vegetative growth stage, represents the second-generation insecticidal toxin. In recent years, significant progress has been made on its structure and action mechanism. However, how it is translocated across the cytoplasmic membrane into the environment remains a mystery. This work demonstrates that Vip3Aa is not secreted by the General Secretion (Sec) System. To reveal the secretory pathway of Vip3A, we purified the membrane vesicles (MVs) of *B. thuringiensis* BMB171 and observed by TEM. The size of MVs was determined by the dynamic light scattering method, and their diameter was approximately 40–200 nm, which is consistent with the vesicles in Gram-negative bacteria. Moreover, Vip3A could be detected in the purified MVs by Western blot, and immunoelectron microscopy reveals Vip3A antibody-coated gold particles located in the MVs. After deleting its signal peptide, chitinase B (ChiB) failed to be secreted. However, the recombinant ChiB, whose signal peptide was substituted with the N-terminal 39 amino acids from Vip3A, was secreted successfully through MVs. Thus, this sequence is proposed as the signal region responsible for vesicle transport. Together, our results revealed for the first time that Vip3Aa is transported to the medium via MVs.

## 1. Introduction

Vegetative insecticidal protein Vip3Aa, secreted by many *Bacillus thuringiensis* (Bt) strains during the vegetative growth stage, shares no sequence and structural homology with known insecticidal crystal proteins (ICPs) [1,2] and represents the second-generation insecticidal toxin. Vip3Aa has broad-spectrum insecticidal activity and a unique mechanism for killing insects [2], which has an excellent control effect on *Spodoptera frugiperda* and other insect pests, such as *Spodoptera exigua, Helicoverpa armigera, Helicoverpa zea, Heliothis virescens,* and *Agrotis ipsilon* [3,4]. More critically, Vip3A shows synergy with some crystal proteins, and no cross-resistance has been observed between these two kinds of proteins so far [5,6]. Consequently, based on the gene-pyramiding strategy, the *vip3A* and the *cry* genes are utilized simultaneously in rice, cotton, and maize for higher efficacy and to delay insect resistance development [7,8].

Vip3A-related research has mainly focused on its architecture and the action mechanism in recent years. The cryo-EM structure reveals that Vip3A is composed of five domains and its molecular architecture is distinctly different from a 3-domains structure of Cry protein [9,10]. This confirms the previous speculation that Cry and Vip3A toxins do not share receptors in the insect midgut due to structural differences [2]. Moreover, the trypsin-activated structure unravels significant conformational changes upon protease digestion in the N-terminal region [10,11], resulting in the reorganization of domain I into a long needle structure of 200 Å, which remains associated with the rest of the protein. A spring-loaded mechanism was proposed to explain its activation process [10].

Binding to the receptor is a crucial step in the virulence process of Vip3A. Recently, several Vip3A receptors have been identified, including ribosome S2 protein from sf21 cells of *S. frugiperda* [12], scavenger receptor class C protein (Sf-SR-C) [13], fibroblast growth factor receptor protein (Sf-FGFR) [14] and prohibitin 2 (PHB2) from the Sf9 cell lines of *S.frugiperda* [15], and a tenascin-like glycoprotein from *A. ipsilon* [16]. After binding to its receptor, Vip3A may exert its potency through pore formation and induce apoptosis [1,17,18]. Although the pore formation model is most acceptable for the activity of Vip3Aa, more and more evidence shows that the induction of apoptosis through an intrinsic mitochondrial pathway may also be involved in its toxicity [19].

Vip3A was first identified as a secreted protein in the supernatant of *Bt* strain AB88 [20]. By sequence alignment, it was found that the N-terminus of Vip3 is highly conserved. However, Vip3A is not N-terminally processed during export, and sequence analysis using SignalP 6.0 (https://services.healthtech.dtu.dk/service.php?SignalP-6.0, accessed on 5 March 2022) reveals that Vip3Aa lacks a classical signal peptide, it is proposed to be secreted through a non-classical secretion pathway. In recent years, significant progress has been made in the structure and mechanism of action of Vip3A, but its secretory mechanism is still undefined. How it translocates across the cytoplasmic membrane remains a mystery.

In this study, we tried to reveal the secretory pathway of Vip3A and define the region responsible for its transport process. We demonstrated that Vip3A is not secreted by General Secretion (Sec) System. Instead, it transports to the medium via membrane vesicles. Furthermore, the N-terminal 39 amino acid sequence of Vip3Aa is proposed as the signal region responsible for vesicle transport, leading ChiB without signal peptides to be secreted by vesicles. These results solve a long-standing question concerning the secretion of the Vip3A, which can improve the secretion of Vip3A and may apply to other proteins.

## 2. Results

### 2.1. Vip3A Is Not Secreted via the Sec Secretion Pathway

**Figure 1:**
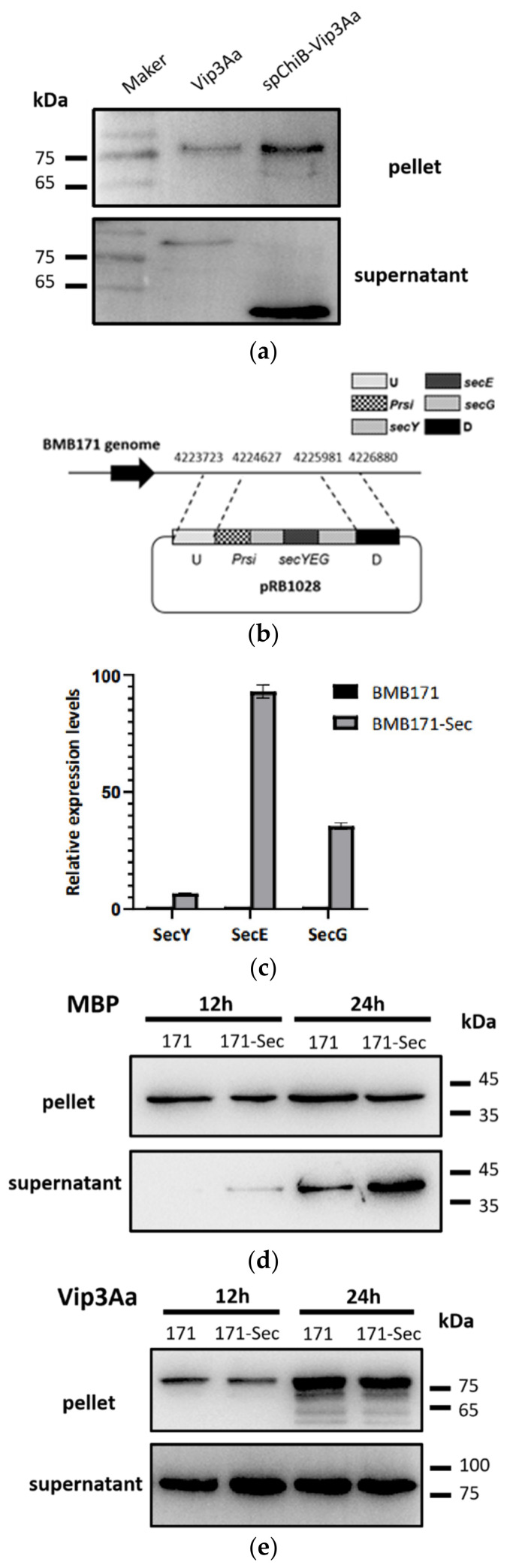
Vip3Aa protein is not secreted via the sec secretion pathway. (**a**) Western blot analysis of Vip3Aa and ChiB signal peptide-guided Vip3Aa in the pellet (upper panel) and the supernatant (lower panel) of BMB171. (**b**) Illustration of how SecYEG-fused protein gene integrated onto the genome of BMB171strain to generate a knock-in strain BMB171-Sec. (**c**) Relative expression levels of SecYEG genes in the BMB171 and BMB171-Sec were determined by qRT-PCR analysis. (**d**,**e**) Western blot analysis of Maltose Binding Protein (MBP) (**d**) and Vip3Aa (**e**) in BMB171 and BMB171-Sec expressed in the pellet (upper panel) and the supernatant (lower panel).

The ChiB of *Bacillus cereus* is secreted by the Sec system [21]. *B. cereus* and *B. thuringiensis* are closely related bacteria of the *B. cereus* group. Previous research in our laboratory indicated that ChiB from *B. thuringiensis* has a predicted Sec signal peptide and translocates by the Sec system (data not shown). To determine whether Vip3Aa is secreted through the Sec pathway, we preliminarily explored the effect of using the Sec pathway signal peptide of ChiB on Vip3Aa secretion efficiency. To express a large amount of Vip3A protein to determine its secretion, we constructed plasmid pPCspvip3. *P_rsi_* is a strong constitutive promoter previously found in our laboratory, and we used it to drive Vip3A expression. Moreover, we used the signal peptide of ChiB to direct the secretion of Vip3A, and Vip3A was detected using an anti-Vip3A antibody. The result showed that although spChiB-Vip3A emerged in the supernatants, contrary to Vip3Aa, much of them have been degraded (Figure 1a). This suggested that when Vip3A secretes through the Sec pathway, it encounters specific proteases that have not been met before. In other words, the ChiB signal peptide transports Vip3A to the culture through a completely different secretory pathway from Vip3A itself.

Additionally, we enhanced the Sec secretion system through overexpression of *secYEG*, which serves as a translocation channel in the Sec secretion system. Under the control of a strong promoter *P_rsi_*_,_ a DNA fragment coding for SecYEG-fused protein was integrated onto the BMB171 genome to generate a knock-in strain BMB171-Sec (Figure 1b). We validated the overexpression of *secYEG* by qRT-PCR (Figure 1c).

ChiB has high secretion efficiency and few intracellular residues in *B. thuringiensis* BMB 171, which is not conducive to verifying the enhancement of the Sec secretion system. In contrast, the secretion efficiency of Maltose Binding Protein (MBP), a classical Sec system secreted protein derived from *Escherichia coli* K-12, is moderate in *B. thuringiensis* BMB 171. We used it to identify whether Sec-dependent protein secretion efficiency has improved. The recombinant plasmid pHT1K-*vip3A* and pHT1K-*mbp* were transformed into the knock-in strain BMB171-Sec, respectively. The results indicated that the secretion efficiency of MBP enhanced significantly in BMB171-Sec (Figure 1d), while the Vip3A content was similar in the supernatant of BMB171 and BMB171-Sec (Figure 1e). This also supported that the Sec system does not secrete Vip3A.

### 2.2. Isolation of Vesicles from B. thuringiensis BMB171 Culture Supernatants

**Figure 2:**
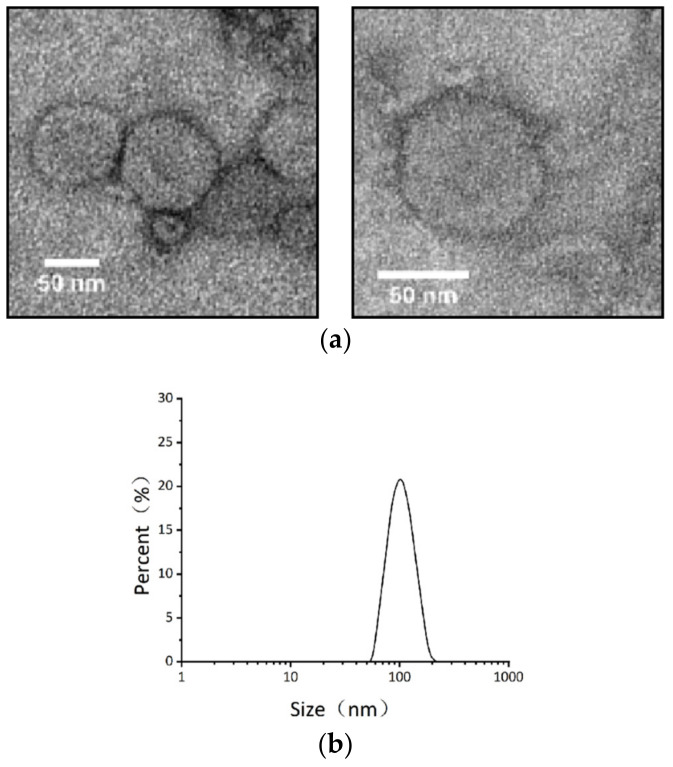
Gram-positive bacteria *B. thuringiensis* BMB171 can produce MVs. (**a**) Negative-staining TEM of purified MVs (scale bars, 50 nm). (**b**) Size distribution of MVs measured with dynamic light scattering (DLS) shows the diameter range of 40–200 nm.

To investigate whether *B. thuringiensis* produces MVs, we collected vesicles in culture supernatants by hypercentrifugation. After negative staining, some cup-shaped structures with a transparent membrane were visualized using a transmission electron microscope (TEM) (Figure 2a), which is similar to the extracellular vesicles figure from Shao ‘s review in 2018 [21].

Dynamic light scattering analysis showed that the diameter of purified MV ranges from 40 to 200 nm (Figure 2b), consistent with that of Gram-negative bacteria outer membrane vesicles.

### 2.3. Vip3Aa Were Detected in Membrane Vesicles

**Figure 3:**
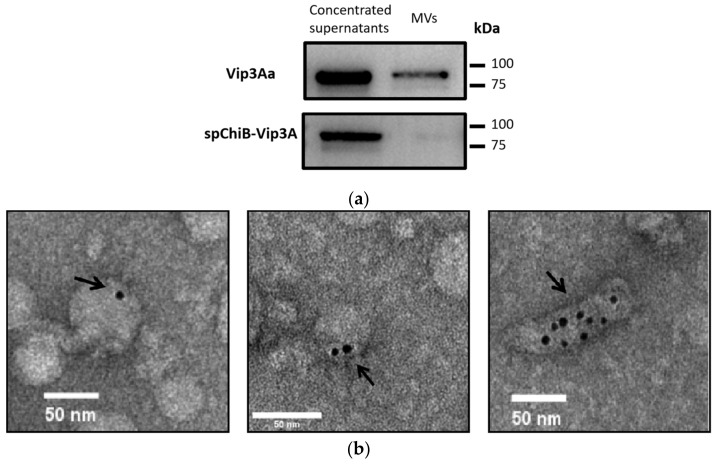
Vip3Aa was detected in membrane vesicles. (**a**) MVs collected from the culture media of BMB171/Vip3Aa and BMB171/ChiBsp-Vip3Aa were analyzed by Western Blot. Samples were detected by anti-Vip3Aa antibody. (**b**) Immunoelectron microscopy revealed Vip3Aa in isolated vesicles. Solid arrows, gold particles (6 nm) depicting Vip3Aa binding (scale bars, 50 nm). After permeabilization, some membrane vesicles structure are disrupted and other morphologies appear.

Vip3Aa can be detected in vesicles purified from the BMB171/ pHT1K-*vip3A* strain by Western blot analysis (Figure 3a). To determine whether Vip3A is present in the vesicles, we performed immunogold electron microscopy (IEM) of purified vesicles (Figure 3b). The result indicated the presence of Vip3A antibody-coated gold particles in purified vesicles, while immunogold labeling with an antibody against ChiB did not reveal gold particles in isolated vesicles.

Additionally, vesicles from the BMB171 in which Vip3A is guided by ChiB signal peptide were also collected. We detected Vip3A in the supernatant but not in purified MVs (Figure 3a). This provided further evidence that the ChiB signal peptide-guided Vip3A protein secretion is distinct from Vip3A.

### 2.4. Vip3Aa α-Helix Plays an Essential Role in Vip3Aa Secretion

**Figure 4:**
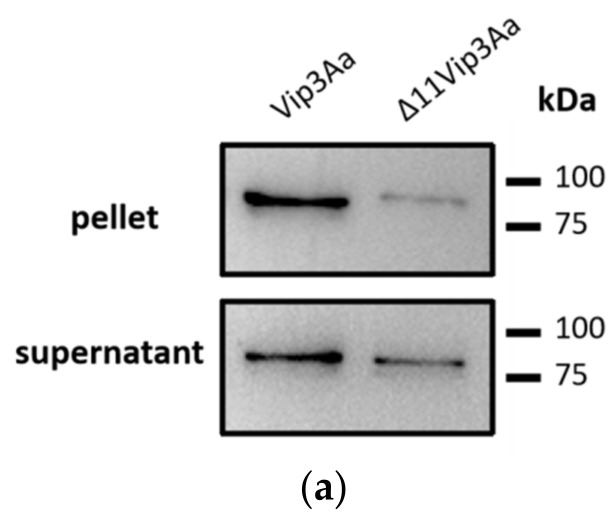

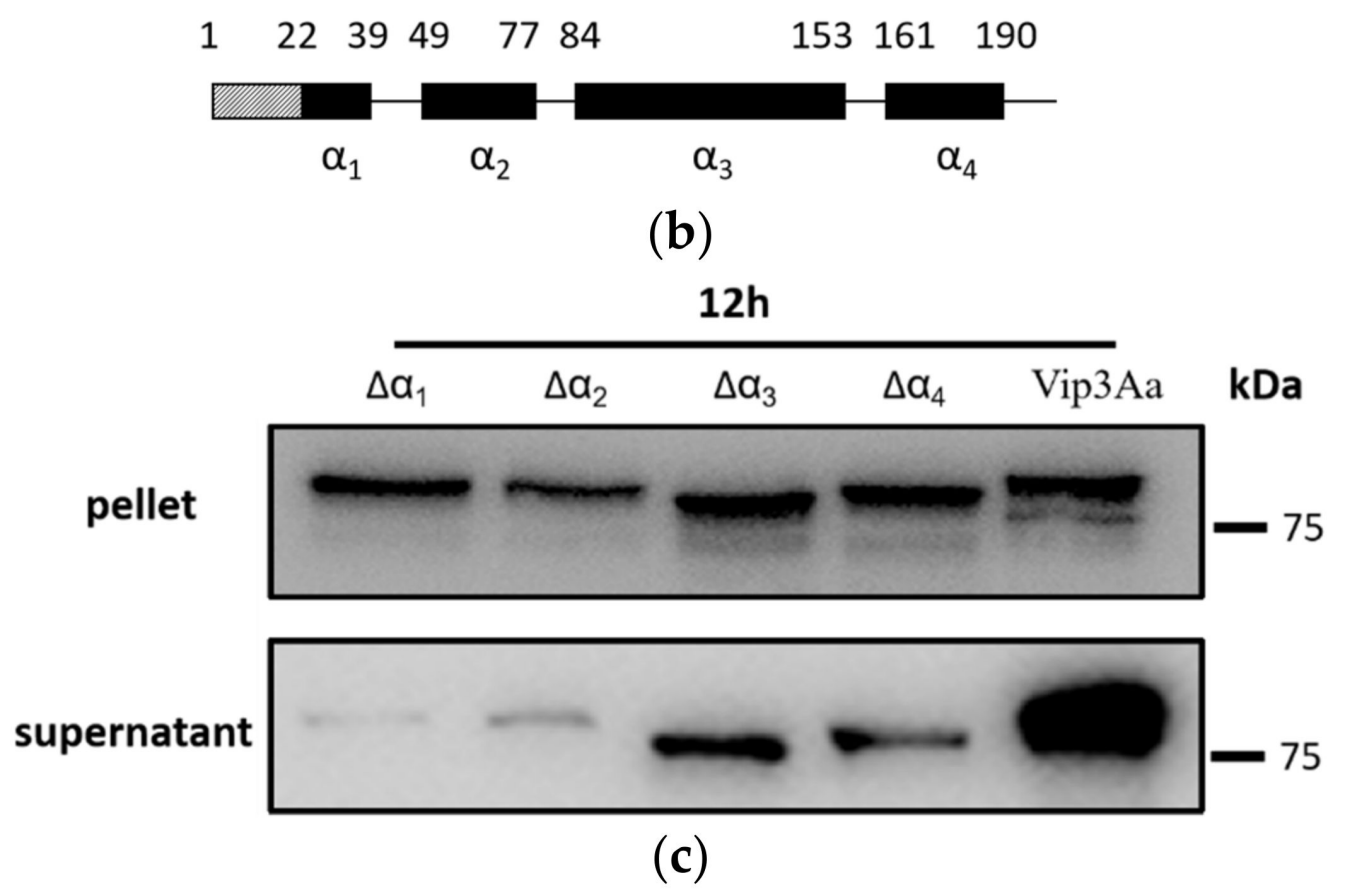
Critical regions of Vip3Aa that affect its secretion. (**a**) Western blot analysis of Vip3Aa and Δ11Vip3Aa expressed in the pellet (upper panel) and the supernatant (lower panel). (**b**) The organization of Vip3Aa DomainI. (**c**) Western blot analysis of Δα_1_-Vip3Aa, Δα_2_-Vip3Aa, Δα_3_-Vip3Aa, Δα_4_-Vip3Aa, and Vip3Aa expressed in different α-helix-deficient strains was visualized in the pellet (upper panel) and the supernatant (lower panel). Samples were collected at 12 h (left) and 24 h (right).

The highly conserved N-terminal region of Vip3A was supposed to be responsible for its translocation and virulence. Zack et al. considered that Vip3A contains a putative signal peptide cleavage site at 12ALPSF [22]. However, using deletion mutagenesis to excise amino acid residues 1–11 of the Vip3Aa, we found that the generated mutant protein can still be secreted into the culture supernatant (Figure 4a).

Proteomics of *B. anthracis* MVs identified thirty-six different proteins [23]. When performing the motif analysis by MEME (http://meme.nbcr.net, accessed on 3 March 2022), we found no conserved sequence among these proteins and Vip3Aa. However, they have similar characteristics in protein secondary structure. The 3D structure of Vip3Aa shows that its N-terminal contains four helices [10]. Similarly, the protein secondary structure predictions indicated that the N-terminus of *B. anthracis* MV proteins had two or more α-helices. (Figure 4b).

To clarify the effect of these α-helices on the secretion of Vip3Aa, we constructed different α-helix-deficient strains. We found that no matter which α-helix was deleted, Vip3Aa can still be detected in the supernatant (Figure 4c). However, the deletion of α_1_ and α_2_ significantly affected the secretion efficiency of Vip3Aa. Thus, α_1_ and α_2_ may serve as sorting signals of vesicular secretory proteins.

### 2.5. The Signal Region of Vip3Aa Can Direct Other Proteins into Vesicles

**Figure 5:**
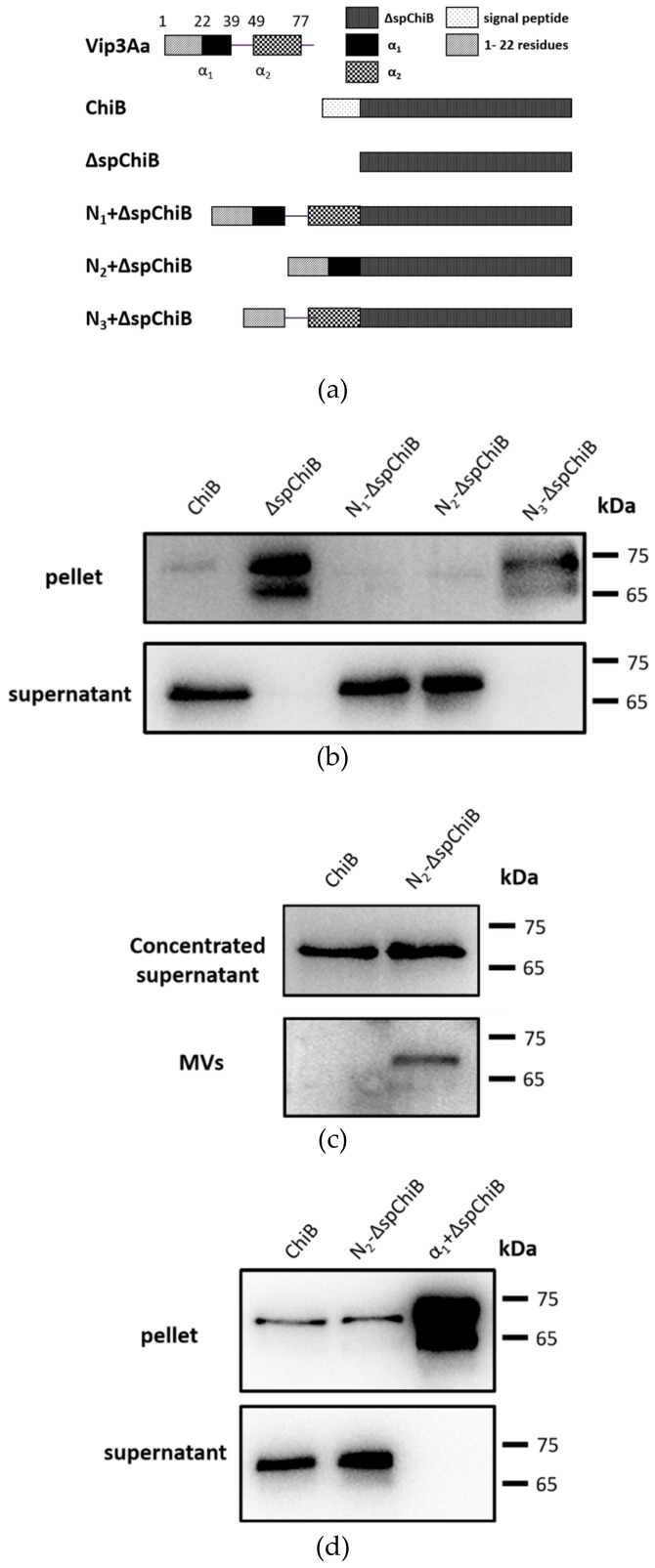
Identification of the signal regions for MV secretion. (**a**) Replacing the *chiB* signal peptide sequence with Vip3A possible signal regions. (**b**) Western blot analysis of ChiB, ΔspChiB, N_1_-ΔspChiB, N_2_-ΔspChiB and N_3_-ΔspChiB expressed in BMB171. Samples were collected at 12 h. (**c**) Concentrated supernatant (upper panel) and MVs (lower panel) purified from the culture media of BMB171/ChiB and BMB171/N2-ΔspChiB were analyzed by Western blotting. (**d**) Western blot analysis of ChiB expressed in BMB171/ChiB, BMB171/N_2_-ΔspChiB and BMB171/α_1_ + ChiB. Samples were collected at 12 h.

When the *chiB* gene without signal peptide sequence was expressed in Bt BMB171, ChiB protein accumulated in the cytoplasm instead of appearing in the culture supernatant. However, when replacing the signal peptide sequence with Vip3A 3 possible signal regions, N_1_ (N-terminal 77 amino acids including α_1_ and α_2_), N_2_ (N_1_ without α_2_) and N_3_ (N_1_ without α_1_), (Figure 5a) ChiB could be detected in the supernatant when α_1_ exists (Figure 5b). Thus, α_1_ appears to play an essential role in protein secretion. Moreover, ChiB was detected in vesicles collected from the supernatant (Figure 5c), suggesting that MVs can transport other secreted proteins if appropriate signaling regions are available.

Contrary to Vip3A, the α_2_-helix is not indispensable for guiding the secretion of ChiB. The N-terminal 39 amino acid sequence of Vip3Aa, which includes α_1_ and the N-terminal 22 amino acids in front of α_1_ (Figure 5c), is enough for ChiB’s MV transport. Consequently, we refer to it as the signal region responsible for MV secretion. In addition, we found when we removed the N-terminal 22 amino acids in front of α1, ChiB accumulated in the intracellular. This suggests that N-terminal 22 amino acids or α1 is indispensable (Figure 5d).

## 3. Discussion

In 1996, Estruch et al. first discovered Vip3A and noticed it is secreted without N-terminal processing [20]. They suggested that a non-classical secretion pathway secretes Vip3Aa. After that, few researchers explored the secretion mode of Vip3A.

At least ten secretion systems (the Sec system, the TAT system, secretion systems I–VII, and outer membrane vesicles) have been identified in Gram-negative bacteria [24,25]. By comparison, much fewer systems are present in Gram-positive bacteria [26]. Type V (T5SS) and type VI (T6SS) secretion systems with antibacterial effects are restricted to Gram-negative bacteria. Type VII secretion systems (T7SSs) in *Mycobacterium tuberculosis* secrete virulence factors belonging to the WXG100 family. In addition, T7SS of Gram-positive bacteria may secrete a class of putative antibacterial toxins [27].

Using the online software signalp-6.0, we found that the N-terminal sequence of Vip3A has no typical Sec and TAT signal peptides features. Moreover, Sec pathway improvement does not facilitate Vip3A secretion in this study. Thus, our results indicated that these two well-documented secretion pathways do not secrete Vip3Aa.

Since 1965, membrane vesicles (MVs) produced by the outer membrane of Gram-negative bacteria were first detected [27], and membrane vesicles have been observed in many bacteria. Vesicles have also been isolated and purified from Gram-positive bacteria over the past two decades [23,26], and some scholars term them CMVs [28], which are derived from cytoplasmic membranes. Although some evidence indicates that vesicles from Gram-negative and Gram-positive share a lot of properties and capabilities [29], vesicle secretion in Gram-positive bacteria is less studied. It seems that it has never been explored as a secretory pathway.

In this study, *B. thuringiensis* MVs were detected in size between 40 and 200 nm, expanding the horizon for MVs in Gram-positive bacteria. More importantly, Vip3A was confirmed to be transported by MVs. To our knowledge, it is the first time to reveal the secretion pathway of Vip3A.

In addition, Wang et al. [30] reported that Vip3Aa secretion is nutrient-dependent. When grown in a sporulation medium, Vip3Aa were not secreted to the culture solution but were retained in the mother cell compartment. However, when the strains were cultured in a rich medium, a fraction of Vip3Aa was secreted. Similarly, it was found that the MV yield of *Pseudomonas putida* KT2440 in LB media was three-fold increased than that of the minimal medium [31]. Given that, we hypothesized that oligonutrition blocks the vesicle formation and further impedes the secretion of Vip3A [32]. Thus, the optimal culture condition needs to be explored to improve MV secretion efficiency.

High concentrations of Vip3A are prone to form tetramers. An essential characteristic of MVs is the enrichment of specific protein and lipid cargo [33]. In this secretion pathway, the presence of vesicles may contribute to the formation of the Vip3A tetramer structure. Vesicles may provide a space for Vip3A, where its monomers can aggregate with each other and avoid being cleaved by proteases due to the protection of the membrane. As with Vip3A, inactive ClyA monomers in *E.coli* are packed into the OMVs and polymerized into a potent toxin in OMVs [34].

The production of extracellular vesicles in bacteria is a well-documented process, but the cargo selection mechanism of MVs is poorly understood [35]. A conserved, negatively charged amino acid motif known as the lipoprotein export signal (LES) domain is required for surface exposure of vesicles lipoproteins in *Bacteroidetes*. The LES domain is the first identified vesicles protein-sorting signal [36].

After determining that MVs secrete Vip3A, we tried to identify the signal region responsible for vesicle transport. For Vip3A, it still be secreted when the α_1_ is removed. One possible explanation is that there are five α helices in Vip3A domain I. When α_1_ is missing, the latter helices may play a complementary role. For ChiB without its signal peptide, our results showed that the N-terminal 39 amino acid sequence of Vip3Aa could efficiently guide it to the culture medium. Consequently, we proposed that the Vip3Aa N-terminal sequence with conservative secondary structure is the signal region for MV sorting, which needs further exploration.

*Bacillus* is an attractive host for producing heterologous proteins, capable of secreting functional extracellular proteins directly to the culture medium [37]. However, some heterologous proteins are degraded by the host proteases during the secretory process, or traditional signal peptides cannot effectively transport these proteins to the medium. Using typical signal peptides to lead Vip3A secretion, we found that the secretion efficiency and protein stability of Vip3A decreased. Instead, when the Vip3A signal region was used to guide the secretion of ChiB, the transport efficiency was increased, accompanying the improvement of protein stability relative to its signal peptide. This may be because the MV membrane structure protects the packed proteins, which confers MV secretion advantages compared with traditional secretion systems.

MVs have potential in medicine, biology, and nanotechnology, such as employed in nanotechnology, delivering antibiotics or anticancer drugs, and killing bacteria [38,39]. They can also be developed into effective vaccines [40,41], and vesicle immunization effectively protects mice from *B. anthracis* [23]. *B. thuringiensis* BMB171 is nonpathogenic and has little impact on human and animal safety. Heterologously expressing toxin in BMB171 using the MV signal region, biomolecules can be delivered and brought together in MVs. Compared with *B. anthracis* MVs, BMB171 MVs undoubtedly have better potential as a vaccine. In addition, they can certainly be used for DNA and RNA transfer as well [42].

## 4. Conclusions

Overall, our results unveiled that the MV pathway secretes Vip3A. Its N-terminal 39 amino acid sequence, including α_1_, is the transport signal region, which can also lead other proteins to be secreted by vesicles. Our results will contribute to increasing the secretory efficiency of Vip3A. Likewise, it provides a new idea for the efficient transport of other proteins and has bright application prospects in the medicine and pharmaceutical field.

## 5. Materials and Methods

### 5.1. Bacterial Strains and Plasmids

The shuttle vector pHT1K was used for constructing protein expression vectors, and pRB1028 was applied for constructing the gene-integrating expression vectors in this study. The bacterial strains and plasmids used in this study are listed in Table 1.

### 5.2. Media and Cultural Conditions

Recombinant *E. coli* strains were cultivated at 37 ℃ in Luria–Bertani (LB) broth (10 g/L NaCl, 10 g/L tryptone, 5 g/L yeast extract, pH 7.0) with shaking at 200 rpm. *B. thuringiensis* were incubated at 28 °C with shaking at 200 rpm. The corresponding titer of antibiotic (50 μg/mL ampicillin, 50 μg/mL erythromycin or 20 μg/mL kanamycin) was added into the medium if necessary.

### 5.3. Gene Integration in B. thuringiensis

The genes *secY*, *secE*, *secG* mediated by *Prsi* promoter were integrated into the genome of *B. thuringiensis* BMB171 to over express *secYEG* according to previously reported method [20,21]. The construction procedure of BMB171-Sec was described briefly as follows. First, the upstream (U), *secY*, *secE*, *secG* and downstream (D) were amplified, respectively, using PCR with *B. thuringiensis* BMB171 genomic DNA as the template, fused by splicing overlap extension (SOE)-PCR using the primers U-F/D-R. The fused fragments were inserted into the pRB1028 at the restriction sites *Hand*III and *Bam*HI by using the pEASY^®^-Basic Seamless Cloning and Assembly Kit (TransGen Biotech, Beijing, China). pRB-secYEG was constructed successfully after DNA sequencing and was electro-transferred into *B. thuringiensis* BMB171. The positive transformants were cultivated in the 5 mL LB liquid medium with 300 μg/mL spectinomycin at 28 °C, 200 r/min for 6–12h, and subcultured for 5–10 generation. Bacteria were streaked onto Spc300-plates and left overnight at 28 °C, and single colonies were picked and cultured in LB liquid for PCR analysis to screen the single-crossover colonies. Following clone examination by bacterial fluid PCR, the positive clones were subcultured in LB liquid medium without spc300 at 37 °C for 8 h, and subcultured for 5–10 generation. Bacteria were streaked onto LB plates and left overnight at 28 °C, and each single colony was inoculated onto LB plate with or without Spc300. The double crossover-colonies can only grow on the LB plate. After DNA sequencing, *secYEG* overexpressed strain (*B. thuringiensis* BMB171-Sec) was constructed successfully.

### 5.4. Construction of the Protein Expression Vector

The Vip3Aa, MBP, ChiB expression vector used in this study was constructed according to the following method. In previous work from our laboratory, we found *Prsi* is a strong constitutive promoter, it was amplified from Bti75. The DNA of vip3Aa, Δα_1_*vip*, Δα_2_*vip*, Δα_3_*vip*, Δα_4_*vip*, N_1_, N_2_ and N_3_ was amplified from the Vip3Aa11 gene (Genbank accession No. AY489126.1). The DNA of *chiB* and Δsp*chiB* was amplified from the plasmid BMB171/chiB stored in lab. The gene *mpb* coding for MBP was amplified from the genome DNA of DH5α. The DNA fragments were purified, respectively, fused by SOE-PCR, and inserted into the expression vector pHT1K at the restriction sites NcoI and KpnI. All plasmids were constructed by using the pEASY^®^-Basic Seamless Cloning and Assembly Kit (TransGen Biotech, Beijing, China), and the reactions system was transformed in to *E. coli* DH5α. After plasmids sequencing, the recombinant plasmids were electro-transferred into the *B, thuringiensis* and verified by diagnostic PCR. All plasmids constructed in this study are listed in Table 1. All the primers used in this study are listed in Supplementary Table S1.

### 5.5. RNA Extraction and qRT-PCR

The total RNA was extracted by using the TRIzol^®^ Reagent (Invitrogen, Waltham, MA, USA) and then quantified using NanoDrop technology (Thermo Scientific, Santa Clara, CA, USA). cDNA was synthesized using a PrimeScript^TM^ RTreagent Kit with gDNA Eraser (Takara, Japan) according to the manufacturer’s instructions. The cDNA was then used as the template for quantitative real-time PCR (qPCR). Quantitative real-time reverse transcription PCR (qRT-PCR) was performed using TB Green^®^ Premix Ex Taq^TM^ II (Takara) and the PCR products were detected with the StepOnePlusReal-Time PCR System (Applied Biosystems, Foster City, CA, USA). The relative expression level of each gene was calculated according to the 2-11CT method using 16S rRNA from *B. thuringiensis* BMB171 as an internal control.

### 5.6. Western Blotting

Pellets were resuspended in lysis buffer, cells were disrupted by sonication and the lysate was clarified by centrifugation. Culture supernatants and lysates were added into 5x Laemmli sample buffer and boiled for 10 min, separated on a 12% SDS/PAGE, and transferred to nitrocellulose membrane. Membrane was blocked for 2 h with non-fat milk and incubated for 1 h with polyclonal antibodies. After washing five times in TBST, the membrane was incubated with secondary antibodies for 1 h and five times washing. Protein bands were visualized using an enhanced chemiluminescence.

### 5.7. Purification of MVs

MVs were purified from *B. thuringiensis* supernatants with some modified purification methods of one-step sucrose cushion ultracentrifugation (SUC) method [45]. Briefly, overnight activated strains were inoculated to 1L of LB to bring its OD600 to 0.1, and were grown for 12 h at 28 °C with gentle shaking (150 rpm). After the cells were pelleted at 10,000× *g* for 20 min, the supernatant was filtered through a 0.45 mm vacuum filter, and the filtrate was concentrated to 70 mL by ultrafiltration with 100 kDa Amicon Ultra centrifugal filter tube (Millipore, Burlington, MA, USA). The concentrated solution was again filtered through a 0.22 mm vacuum filter. The filtrate was loaded over 4 mL of 30% sucrose solution (in 1 × PBS) and centrifuged at 150,000× *g* for 1.5 h at 4 °C. The pellets were resuspended in PBS and washed by ultracentrifugation at 150,000× *g* for 1.5 h at 4 °C. MVs were resuspended in 200 μL 1 × PBS and stored at −80 °C until use. DLS measurement was performed to measure the diameter of MVs using a DLS analyzer.

### 5.8. TEM Analysis

MV suspension was mixed with an equal volume of 4% PFA. Sample was placed in Formvar-carbon-coated copper grids and allowed to adsorb for 20 min in dry environment. Grids are washed with PBS and transferred to a 50-µL drop of 1% glutaraldehyde for 2 min. Then, transfer to a 100-µL drop of distilled water for 2 min. Repeat seven times. Transfer grids to a 20-µL drop of 2% uranium acetate solution for 2 min. After this, air dry the grid and observe it under the transmission electron microscope (TEM) (HT7700, Tokyo, Japan).

### 5.9. IEM Analysis

MV suspension was mixed with an equal volume of 4% PFA. Sample was placed in Formvar-carbon-coated copper grids and allowed to adsorb for 20 min in dry environment. Transfer grid to PBS and wash twice for 3 min. Permeabilization was performed with 0.1% sapon for 30 min. Transfer grid to PBS/50 mM glycine for 3 min. Transfer grids to a drop of 5%BSA for 10 min to blocking. Transfer the grid to a drop of diluted first antibody and incubate 30 min. Transfer grids to the appropriate washing buffer and wash 3 min. Repeat transfers to drops of washing buffer for a total of six washes. Transfer grids to a drop of diluted 6-nm colloidal gold-labeled secondary antibody, and incubate 30 min. Transfer grids to drops of PBS/0.5% BSA for a total of six washes. Transfer grids to drops of PBS and wash 2 min for 6 times. Transfer grids to 50-µL drops of 1% glutaraldehyde for 2 min. Then, transfer to a 100-µL drop of distilled water for 2 min. Repeat seven times. Transfer grids to a 20-µL drop of 2% uranium acetate solution for 2 min. After this, air dry the grid and observe it under the electron microscope.

## Figures and Tables

**Table 1:** Bacterial strains and plasmids used in this study.

Strains and Plasmids	Relevant Properties	Source of Reference
*Escherichia coli*		
DH5α	F- φ80 *lac* ZΔM15 Δ (*lac*ZYA-*arg* F) U169 *end*A1 *rec*A1 *hsd*R17(*r_k_^−^, m_k_^+^*) *sup*E44λ-*thi*-1 *gyr*A96 *rel*A1*pho*A; host strain for plasmid construction	Stored in lab
*Bacillus thuringiensis*		
BMB171	An acrystalliferous mutant strain; high transformation frequency	[43]
BMB171-Sec	*secYEG* integrate into BMB171 genome, overexpression of *secYEG* in BMB171	This study
BMB171/pHT-*vip3Aa*	BMB171 harboring pHT-*vip3Aa*	This study
BMB171/pPCsp*vip3*	BMB171 harboring pPCsp*vip3*	This study
BMB171-Sec/pHT-*vip3Aa*	BMB171-Sec harboringpHT-*vip3Aa*	This study
BMB171/pHT-*mbp*	BMB171 harboring pHT-*mbp*	This study
BMB171-Sec/pHT-*mbp*	BMB171-Sec harboring pHT-*mbp*	This study
BMB171/pPΔα_1_*vip*	BMB171 harboring pPΔα_1_*vip*	This study
BMB171/pPΔα_2_*vip*	BMB171 harboring pPΔα_2_*vip*	This study
BMB171/pPΔα_3_*vip*	BMB171 harboring pPΔα_3_*vip*	This study
BMB171/pPΔα_4_*vip*	BMB171 harboring pPΔα_4_*vip*	This study
BMB171/pHT-*chiB*	BMB171 harboringpHT-*chiB*	This study
BMB171/pHT-Δsp*chiB*	BMB171 harboring pHT-Δsp*chiB*	This study
BMB171/pPN_1_Δsp*chiB*	BMB171 harboring pPN_1_Δsp*chiB*	This study
BMB171/pPN_2_Δsp*chiB*	BMB171 harboring pPN_2_Δsp*chiB*	This study
BMB171/pPN_3_Δsp*chiB*	BMB171 harboring pPN_3_Δsp*chiB*	This study
BMB171/pPα_1_VΔsp*chiB*	BMB171 harboring pPα_1_VΔsp*chiB*	This study
*Plasmids*		
pHT1k	*E.coli* and *B.* *thuringiensis* shuttle vector; Amp^R^, Erm^R^	[44]
pHT-*vip3Aa*	pHT1K + Promotor-*Prsi* + *vip3Aa*	This study
pPCsp*vip3*	pHT1K + Promotor-*Prsi* + signal peptide of ChiB + *vip3Aa*	This study
pHT-*mbp*	pHT1K + Promotor-*Prsi* + *mbp*	This study
pPΔα_1_*vip*	pHT1K + Promotor-*Prsi* + Δα_1_*vip3Aa*	This study
pPΔα_2_*vip*	pHT1K + Promotor-*Prsi* + Δα_2_*vip3Aa*	This study
pPΔα_3_*vip*	pHT1K + Promotor-*Prsi* + Δα_3_*vip3Aa*	This study
pPΔα_4_*vip*	pHT1K + Promotor-*Prsi* + Δα_4_*vip3Aa*	This study
pHT-*chiB*	pHT1K + Promotor-*Prsi* + *chiB*	This study
pHT-Δsp*chiB*	pHT1K + Promotor-*Prsi* + Δsp*chiB*	This study
pPN_1_Δsp*chiB*	pHT1K + Promotor-*Prsi* + N_1_ (N-terminal 77 amino acids of Vip3Aa including α1 and α2) + Δsp*chiB*	This study
pPN_2_Δsp*chiB*	pHT1K + Promotor-*Prsi* + N_2_ (N-terminal 39 amino acids of Vip3Aa including α1) + Δsp*chiB*	This study
pPN_3_Δsp*chiB*	pHT1K + Promotor-*Prsi* + N_3_ (N-terminal 22 amino acids and α2 of Vip3Aa) + Δsp*chiB*	This study
pPα_1_VΔsp*chiB*	pHT1K + Promotor*-Prsi* + α1 of Vip3Aa + Δsp*chi*B	This study
pRB1028	*B. thuringiensis* knockout vector; spc^R^	Stored in lab
pRB-*secYEG*	pRB1028-secYEG(U + *secYEG* + D); to overexpress *secYEG*	This study

AmpR, ampicillin resistance; EmrR, erythromycin resistance; SpcR, spectinomycin resistance.

## Supplementary Materials

The following supporting information can be downloaded at: https://www.mdpi.com/article/10.3390/toxins14070480/s1, Table S1: Oligonucleotides used in this study.
