# Supplementary material for: Vegetative Insecticidal Protein Vip3Aa Is Transported via Membrane Vesicles in Bacillus thuringiensis BMB171
Source: Toxins (Basel). 2022 Jul 13;14(7):480. doi: 10.3390/toxins14070480 (PMC9319297; doi:10.3390/toxins14070480)
Supplement: Supplementary file 1 [file toxins-14-00480-s001.zip › toxins-1788579-supplementary.pdf]

Table S1. Oligonucleotides used in this study.

Name	Sequence
vip3A-F	TAACTAACTAAGGAGGAAAATTCATGAACAAGAATAATACTAAATTAAGC
vip3A-R	CGATATTTAAACCTGAGTTTGCATGGTACCTTACTTAATAGAGACATCG
Prsi-F	CGATATTTAAACCTGAGTTTGCATGGTACCTTACTTAATAGAGACATCG
Prsi-R	GAATTTTCCTCCTTAGTTAGTTAAGATG
mbp-F	TAACTAACTAAGGAGGAAAATTCATGAAAATAAAAACAGGTGC
mbp-R	TATTTAAACCTGAGTTTGCATGGTACCTTACTTGGTGATACGAGTCTGC
Csp-vip-F	TAACTAACTAAGGAGGAAAATTCATGAGGTCTCAAAAATTCACAC
Csp-vip-R	CTTAATTTAGTATTATTCTTGTTCATTGCTAATGCGAGATTTGGAG
$\Delta\alpha_1$Vip-F	AACTAACTAAGGAGGAAAATTCATGATTTATGGATTTGCCAC
$\Delta\alpha_2$Vip-F	AACTAACTAAGGAGGAAAATTCATGGACGAAATTTTAAAGAATC
$\Delta\alpha_3$Vip-F	AACTAACTAAGGAGGAAAATTCATGGAATTATCTAAGGAAATATTA
$\Delta\alpha_4$Vip-F	AACTAACTAAGGAGGAAAATTCATGCTTATTAACTCTACACTTAC
N1-R	CTTTGCTTTGGTGAATCTGCGATAAGATCATTTAAGC
N2-R	TTTGACTTTGCTTTGGTGAATCTTTAAAAATCATGTTCATAATG
chiB-F	TAACTAACTAAGGAGGAAAATTCATGAGGTCTCAAAAATTCACAC
chiB-R	TTAAACCTGAGTTTGCATGGTACCCTAGTTTTCGCTAATGACGGC
ΔspchiB-F	TTTGACTTTGCTTTGGTGAATC
$\alpha_1$chiB-F1	CTGGTATCAAAGACATTATGAACATGATTTTTAAAGATTCACCAAAGCAAAG
$\alpha_1$chiB-F2	ACTAACTAAGGAGGAAAATTCATTTATGGATTTGCCACTGGTATCAAAGACA
secY-F	GAAAGGAGGAATTCATCTAATTGAAGCAGCTGGCGGATCAG
secY-R	GTGTATTATTTCAATTATTTATTGATAAACCCTTTGTAATGG
secE-F	CCATTACAAAGGGTTTATCAATAAATAATTGAAATAATACACTG
secE-R	ATTCAGTTATTGTAACTGTAGCATTTAACCAAGAATTAACC
secG-F	CGGTTAATTCTTGGTTAAATGCTACAGTTACAATAACTGAATC
secG-R	ACGTTCTTTCGTAACTAAATCTATAAGTTCAAGTACGTAACA
U-F	GTCGGGAATTGATGCAGAAGCTTATTAGGGGGAGTATCAATG
U-R	CTTTTTGACAACTTACCCTATCAAGGGAGAGGTTTAACAC
D-F	GTTACGTACTTGAACTTATAGATTTAGTTACGAAAGAACGTG
D-R	GGTACCATGCATGTCGACGGATCCCCACTCACTTTACGATAACG
16s-F	GTGATGAAGGCTTTCGGGTCGT
16s-R	CACTCAAGTCTCCCAGTTTCCATG
secYs-F	ATGCGAAGCGTGTAACAGGA
secYs-R	GTGCAATAGTCGGAGGCGTA
secEs-F	TTACTCCGCTCAACAGCGAC
secEs-R	ACCGCGAAGAAAATCGCAAA
secGs-F	ATCCCACGTGCTTTTTGCTT
secGs-R	TCAGGCCTTTCAGGTGCAAT
